# An online atlas of human plasma metabolite signatures of gut microbiome composition

**DOI:** 10.1038/s41467-022-33050-0

**Published:** 2022-09-23

**Authors:** Koen F. Dekkers, Sergi Sayols-Baixeras, Gabriel Baldanzi, Christoph Nowak, Ulf Hammar, Diem Nguyen, Georgios Varotsis, Louise Brunkwall, Nynne Nielsen, Aron C. Eklund, Jacob Bak Holm, H. Bjørn Nielsen, Filip Ottosson, Yi-Ting Lin, Shafqat Ahmad, Lars Lind, Johan Sundström, Gunnar Engström, J. Gustav Smith, Johan Ärnlöv, Marju Orho-Melander, Tove Fall

**Affiliations:** 1 Department of Medical Sciences, Molecular Epidemiology and Science for Life Laboratory, Uppsala University, Uppsala, Sweden; 2 CIBER Cardiovascular diseases (CIBERCV), Instituto de Salud Carlos III, Madrid, Spain; 3 Division of Family Medicine and Primary Care, Department of Neurobiology, Care Science and Society, Karolinska Institute, Huddinge, Sweden; 4 Department of Clinical Sciences, Lund University, Malmö, Sweden; 5 Clinical Microbiomics A/S, Copenhagen, Denmark; 6 Department of Medical Sciences, Uppsala University, Uppsala, Sweden; 7 Department of Medical Sciences, Clinical Epidemiology, Uppsala University, Uppsala, Sweden; 8 The George Institute for Global Health, University of New South Wales, Sydney, Australia; 9 The Wallenberg Laboratory/Department of Molecular and Clinical Medicine, Institute of Medicine, Gothenburg University and the Department of Cardiology, Sahlgrenska University Hospital, Gothenburg, Sweden; 10 Department of Cardiology, Clinical Sciences, Lund University and Skåne University Hospital, Lund, Sweden; 11 Wallenberg Center for Molecular Medicine and Lund University Diabetes Center, Lund University, Lund, Sweden; 12 School of Health and Social Studies, Dalarna University, Falun, Sweden

**Keywords:** Metabolomics, Microbial genetics

## Abstract

Human gut microbiota produce a variety of molecules, some of which enter the bloodstream and impact health. Conversely, dietary or pharmacological compounds may affect the microbiota before entering the circulation. Characterization of these interactions is an important step towards understanding the effects of the gut microbiota on health. In this cross-sectional study, we used deep metagenomic sequencing and ultra-high-performance liquid chromatography linked to mass spectrometry for a detailed characterization of the gut microbiota and plasma metabolome, respectively, of 8583 participants invited at age 50 to 64 from the population-based Swedish CArdioPulmonary bioImage Study. Here, we find that the gut microbiota explain up to 46% of the variance of individual plasma metabolites and we present 997 associations between alpha diversity and plasma metabolites and 546,819 associations between specific gut metagenomic species and plasma metabolites in an online atlas (https://gutsyatlas.serve.scilifelab.se/). We exemplify the potential of this resource by presenting novel associations between dietary factors and oral medication with the gut microbiome, and microbial species strongly associated with the uremic toxin *p*-cresol sulfate. This resource can be used as the basis for targeted studies of perturbation of specific metabolites and for identification of candidate plasma biomarkers of gut microbiota composition.

## Abstract (web summary)

Here, Dekkers *et* al. characterize associations of 1528 gut metagenomic species with the plasma metabolome in 8583 participants of the SCAPIS Study, and find that gut microbiota explain up to 58% of the variance of individual plasma metabolites.

## Introduction

The bacteria, archaea, viruses, protozoa, and fungi that reside in the gastrointestinal tract are collectively referred to as the gut microbiota. The gut microbiota is shaped by all lifetime exposures of an individual including diet, disease history, antibiotics, and other medication^1^; and by intrinsic factors, such as age and host genetic variation^2^. Conversely, observational studies suggest a role of gut microbiota composition in chronic disease development e.g. cardiovascular disease, obesity, and type 2 diabetes, but evidence of causality and mechanistic understanding of these effects are largely absent^3–5^. Modification of the composition of small molecules in plasma, i.e. the plasma metabolome^3^, has been suggested as a potential mediator of gut microbiota effects on human health, as gut microbiota produce and modify a number of molecules, some of which are taken up into the bloodstream. Consequently, characterization of the interactions between gut microbiota and host plasma metabolites could provide crucial insights into the effects of the gut microbiota on human health.

Previous studies^6–13^ reporting associations between the gut microbiota and the circulating metabolome have been hampered by either small sample size (e.g. <1000 samples), limited data on health-related traits, or limited resolution of gut microbiota composition (e.g. 16S rRNA sequencing) and metabolome data (e.g. NMR profiling). While these studies have shown that the gut microbiota composition is associated with at least a portion of the plasma metabolome, major questions remain. Specifically, since statistical power has been limited, moderate effect sizes or associations of rare species with metabolites have not been possible to assess. Further, there is an imminent need for a public resource of these associations as a useful tool to help the researchers better understand the gut microbiota - plasma metabolome interplay.

Here, we applied state-of-the-art high resolution deep metagenomic sequencing and mass spectrometry-based metabolite profiling to analyze samples from 8583 individuals from SCAPIS, a well-characterized population-based study. We generated the searchable GUTSY Atlas (https://gutsyatlas.serve.scilifelab.se/) of robust associations between the gut microbiota and host plasma metabolome including functional metabolic modules.

## Results

### Gut microbial species and plasma metabolite profiling of the SCAPIS study

SCAPIS^14^ is a prospective population-based observational study of 30,154 men and women living in six municipality regions in Sweden. A randomly selected sample of individuals aged 50 to 64 based on the population register were invited during the years 2014 to 2018 to participate in the baseline investigation. We focused on data and samples obtained at two study sites, Malmö and Uppsala, where fecal samples were collected at home, and from which DNA was successfully extracted, whole-genome shotgun-sequenced, and taxonomically and functionally profiled in 9818 samples. In addition, 8957 fasting venous plasma samples collected during study site visit were successfully profiled using ultra-high-performance liquid chromatography linked to mass spectrometry. Overall, data for 8583 participants that had high quality metagenomics and metabolomics data as well as complete information on main model covariates were used for all analyses. The taxonomic profiling resulted, at the super kingdom level, in 1520 bacterial, 4 archaeal, 2 eukaryotic, and 2 unclassified metagenomic species, from now on called species, which were identified based on their microbial gene profile, with an average of 325 species per sample (range: 26–663, Supplementary Data 1). The metabolite profiling provided data on 1321 metabolites, of which 1052 were annotated from 114 subclasses of metabolites, with an average of 1153 measured metabolites per sample (range: 982 to 1254, Supplementary Data 2). The main sociodemographic and clinical characteristics of these 8583 participants are shown in Table 1. The characteristics of the present study sample were similar to those of the complete study sites of SCAPIS-Uppsala and SCAPIS-Malmö. However, since the study sample for metabolomics was enriched for participants with complete data, there were fewer participants who had missing lifestyle information in the Malmö subsample compared with the complete SCAPIS-Malmö study sample (Supplementary Data 3).

**Table 1:** Main sociodemographic and clinical characteristics of the Malmö and Uppsala SCAPIS study sites included in the current study

	Malmö(*n* = 3811)	Uppsala(*n* = 4772)
Age	57.4 (4.3)	57.7 (4.4)
Sex: female	2009 (52.7%)	2451 (51.4%)
*Place of birth*		
Scandinavia	2976 (78.1%)	4295 (90.0%)
Non-Scandinavian Europe	543 (14.2%)	184 (3.9%)
Asia	202 (5.3%)	193 (4.0%)
Other	90 (2.4%)	100 (2.1%)
Body mass index, kg/m^2^	27.2 (4.5)	27.0 (4.4)
Systolic blood pressure, mmHg	122 (16.4)	125 (15.9)
Estimated glomerular filtration rate	84.5 (12.1)	87.0 (11.5)
*Smoking status*		
Current	640 (16.8%)	431 (9.0%)
Former	1460 (38.3%)	1461 (30.6%)
Never	1678 (44.0%)	2638 (55.3%)
Missing	33 (0.9%)	242 (5.1%)
Fiber intake, g/kcal^a^	0.012 (0.004)	0.012 (0.004)
*Coffee intake*		
<1 times/d	518 (13.6%)	580 (12.2%)
1–2 times/d	1291 (33.9%)	1384 (29.0%)
3–4 times/d	1431 (37.5%)	2116 (44.3%)
>4 times/d	548 (14.4%)	672 (14.1%)
Missing	23 (0.6%)	20 (0.4%)
Any antibiotics, last 12 months	786 (20.6%)	896 (18.8%)
Hypertension medication^b^	775 (20.3%)	883 (18.5%)
Cholesterol medication^b^	319 (8.4%)	352 (7.4%)
Diabetes medication^b^	170 (4.5%)	161 (3.4%)
Dispensed prescription for metformin, last 12 months	163 (4.3%)	143 (3.0%)
Dispensed prescription for omeprazole, last 12 months	452 (11.9%)	396 (8.3%)

Continuous variables are provided as mean (standard deviation) and categorical variables as *n* (%).

^a^Fiber intake, adjusted for total energy intake.

^b^Self-reported medication last 2 weeks.

### Metabolite signatures of microbial diversity

**Fig. 1:**
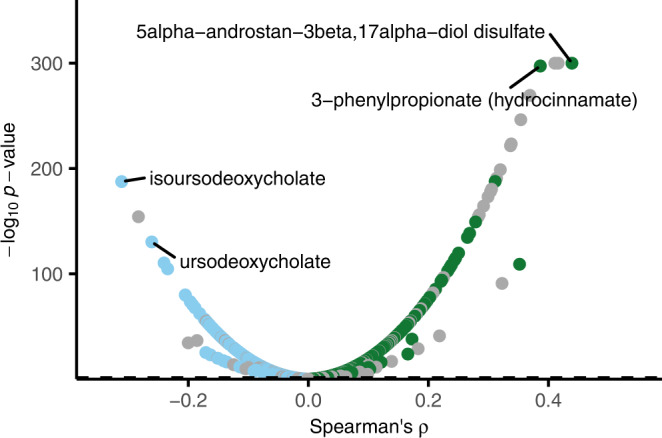
Partial Spearman’s rank correlation between species alpha diversity and 1321 plasma metabolites adjusted for age, sex, place of birth, study site, microbial DNA extraction plate, and metabolomics delivery batch. The association of Shannon diversity index based on deep metagenomic sequencing of fecal samples and 1321 plasma metabolites measured with ultra-high performance liquid chromatography linked to mass spectrometry in 8583 participants aged 50 to 65 of the Swedish CArdioPulmonary bioImage Study. There were 565 significant positive associations and 432 significant negative associations after adjusting for multiple testing using Benjamini-Hochberg’s method at 5% false discovery rate. Green, positive associations; blue, negative associations; gray, indicates the non-characterized metabolites. Labels are shown for the 2 most positively and negatively correlated characterized metabolites. The dashed line represents the multiple testing threshold. The *p*-values were capped at 10^−300^. Source data are provided as a Source Data file.

We first investigated the association of microbial alpha diversity with individual plasma metabolites. Alpha diversity was estimated using the Shannon diversity index, a measure of overall microbiota richness and evenness previously reported as inversely associated with markers of metabolic health^15^. We observed that alpha diversity was positively associated with 565, and negatively associated with 432, of the 1321 plasma metabolites in models adjusted for age, sex, place of birth, study site, microbial DNA extraction plate, and metabolomics delivery batch (Fig. 1 and Supplementary Data 4). There were 109 associations with an absolute Spearman’s *ρ* > 0.15 and 17 associations with an absolute Spearman’s *ρ* > 0.30. Significance was based on *p*-values adjusted for multiple testing, which we report as *q*-values, using the Benjamini–Hochberg method^16^ at a 5% false discovery rate. Regarding annotated metabolites, we observed the strongest positive associations for the metabolite 5alpha-androstan-3beta,17alpha-diol disulfate (*ρ* = 0.44, *p*-value < 10^−300^), a sulfated steroid; and 3-phenylpropionate (hydrocinnamate) one of the main phenolic metabolites present in human feces^17^ (*ρ* = 0.39, *p*-value = 4.0 × 10^−^^298^), and cinnamoylglycine (*ρ* = 0.39, *p*-value = 5.6 × 10^−^^298^). All three are previously reported strongly positively associated with alpha diversity^18^ and the two latter with lower risk of type 2 diabetes^19^. These observations indicate that gut microbial diversity is robustly associated with a range of specific plasma metabolites and motivated the ensuing detailed investigations of specific gut microbiota species.

### Associations of gut microbiota with plasma metabolome show large variation across groups of microbial species and metabolites

**Fig. 2:**
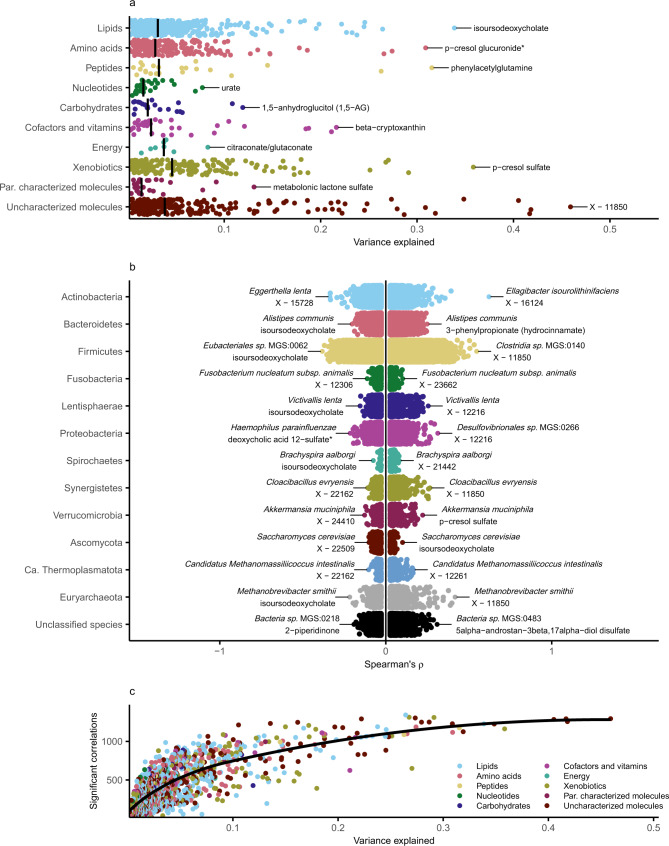
Associations of gut microbiota with plasma metabolome show great variation across groups of species and metabolites. **a** The variance in 1168 of the 1321 metabolites partly explained by variation in the gut microbiota from 8583 individuals aged 50 to 65 of the Swedish CArdioPulmonary bioImage Study. Models were fitted for each metabolite using ridge regression using nested 10-fold cross-validation. The variance explained was calculated as the cross-validated *r*^2^ statistic. Metabolites were grouped by metabolic pathway and the vertical line represents the median of the variance explained for each group. The metabolite with the largest variance explained for each group is annotated. **b** Partial Spearman’s rank correlations between 1528 gut microbial species and 1321 plasma metabolites adjusted for age, sex, place of birth, study site, microbial DNA extraction plate, and metabolomics delivery batch. Depicted are the Spearman’s ρ for 298,982 significant positive associations and 247,837 significant negative associations after adjusting for multiple testing using Benjamini–Hochberg’s method at 5% false discovery rate. Associations were grouped by taxonomic phylum. **c** Variance explained versus number of associated species for 1321 plasma metabolites. Metabolites were grouped by metabolic class. Shown in black is the locally estimated scatterplot smoothing line. Source data are provided as a Source Data file.

We used a series of nested cross-validated ridge regression models to assess the variance in each plasma metabolite explained by the variation of the gut microbiota. We observed that the variance of 1168 of the 1321 metabolites was partly explained by variation in the gut microbiota (Fig. 2a and Supplementary Data 5). We detected the largest variance explained (46%) for an uncharacterized common metabolite with the provisional identifier X-11850. The main feature mass-to-charge ratio (m/z), retention-time index (RI), and measurement platform for all uncharacterized and characterized metabolites are reported in Supplementary Data 2. However, MS/MS spectral data are not shared by the external laboratory (See Data Availability Statement). The variance explained by gut microbiota species was >10% for 133 metabolites and >25% for 22 metabolites, such as uremic toxin *p*-cresol sulfate (*r*^*2*^ = 36%) and the coffee metabolite quinate (*r*^*2*^ = 27%). For trimethylamine N-oxide, TMAO, the end-product of diet-microbiota interaction, which has been suggested involved in cardiovascular and kidney disease pathogenesis^20^, we found a rather low variance explained by the gut microbiota (*r*^2^ = 1.7%). These observations highlight the large heterogeneity in associations of gut microbiota composition with plasma metabolites.

### Associations of gut microbiota with plasma metabolome are many and robust over a range of lifestyle and health factors

We next assessed the links between 1528 microbial species and 1321 metabolites using a series of partial Spearman’s rank correlations, adjusting for age, sex, place of birth, study site, microbial DNA extraction plate, and metabolomics delivery batch. We identified significant associations (*q*-value < 0.05) in 546,819 (27%) of all tested species–metabolite pairs, of which 298,982 were in the positive direction and 247,837 in the negative direction (Fig. 2b and Supplementary Data 6). There were 10,965 associations with an absolute Spearman’s *ρ* > 0.15 and 439 associations with an absolute Spearman’s *ρ* > 0.30. In Supplementary Data 6 we also report results from a sensitivity analysis, where we additionally adjusted for the Shannon diversity index as a measure of alpha diversity. This analysis is potentially less susceptible to false positives due to the compositionality of the microbial species data, at the cost of increased risk of model misspecification and bias, i.e. alpha diversity can potentially act as a mediator or collider. Additionally, adjustment for alpha diversity can result in reduced power due to collinearity of microbial species with alpha diversity. While all species and metabolites had at least one observed association, many species (*n* = 623) were associated with a broad range (>30%) of metabolites. Conversely, 504 metabolites were associated with a broad range (>30%) of species. The associations between species and metabolites were not generally affected by stratification at the tertiles of body mass index (BMI), systolic blood pressure, estimated glomerular filtration rate^21^ (eGFR, a measure of kidney function), fiber intake, nor by exclusion of smokers and individuals who had been prescribed antibiotics within a year of sampling or taken medication for hypertension, dyslipidemia and/or diabetes (Pearson correlation of Spearman’s *ρ* from non-stratified vs stratified models *r* > 0.91, Supplementary Fig. 1), findings which alleviated concerns about major confounding effects by these factors. However, we did find lower correlation of results within participants with low (<1 times per day, *r* = 0.87) or high coffee intake (>4 times per day, *r* = 0.87), current smokers (*r* = 0.84), and participants with cholesterol (*r* = 0.81) or diabetes medication (*r* = 0.64). This could be explained by lower precision of the estimates as groups were smaller (*n*: 331−1220), or by effect modification by these factors for some associations. Generally, metabolites for which the variance explained by gut microbiota was high also had a high number of associations with individual species (Fig. 2c). Overall, these observations show a plethora of associations between gut microbiota species and the metabolome that are in general robust over a range of lifestyle and health factors.

### Certain species are associated with multiple metabolites, often within the same class of metabolites

We observed several examples of the same microbial species having both strong positive and negative associations with metabolites within the same subclass of metabolites, indicating that these species might affect specific processes (Fig. 2b). One observed example is *Haemophilus parainfluenzae*, a bacterial species previously linked to bile tract infections^22^, that was strongly positively associated with the primary bile acid salt cholate and negatively associated with the secondary bile acid deoxycholic acid 12-sulfate. To investigate whether this was a more general pattern, we grouped single metabolites into metabolite subclasses, and assessed whether alpha diversity and individual species were linked to several metabolites of the same subclass, where each subclass contained at least 5 metabolites. Overall, we found evidence supporting the enrichment of two specific metabolite subclasses for alpha diversity (positive: vitamin A metabolism, negative: secondary bile acid metabolism, *q-*value < 0.05, Supplementary Data 7) and of 59 unique metabolite subclasses for 1402 microbial species (3505 total enrichments, *q*-value < 0.05, Supplementary Data 8). Among the 10 strongest enrichments, we observed nine enrichments for secondary bile acids in the species-metabolome associations (positive: *Eubacteriales sp*. MGS:0662, *Enterocloster asparagiformis*, *Oscillospiraceae sp*. MGS:0104, *Anaerotignum faecicola*; negative: *Intestinibacter bartlettii*, *Intestinibacter sp*. MGS:0194, *Turicibacter sanguinis*, *Clostridium disporicum* and *Peptostreptococcaceae sp*. MGS:0200). Given these findings, we set out to leverage the atlas to identify species influencing the rate of deoxycholic acid formation from cholic acid by 7α-dehydroxylation, which is one of the main first steps in the formation of secondary bile acids from primary bile acids. Previous research has detected bile acid 7α-dehydroxylation activity in a limited group of bacteria in the clostridial rRNA cluster XIVa in the *Lachnospiraceae* family in the Eubacteriales order^23^. To identify potential new species with 7α-dehydroxylation activity, we assessed gut species correlated with low plasma levels of the precursor cholic acid and increased levels of the product deoxycholic acid. Both the primary bile acid cholic acid (denoted cholate in the atlas) and the secondary bile acid deoxycholic acid (deoxycholate) had a high variance explained by the microbiota (*r*^2^ = 18% and 22%, respectively), indicating a strong impact of the variation in microbiota composition. Of the 20 species with strongest negative correlation with cholate and 20 species with strongest positive correlation with deoxycholate, seven were in common. We confirmed these findings using a model with deoxycholate/cholate ratio as outcome (all *p*-values < 10^−47^). These species were all from the Eubacteriales order and include novel findings indicating a role in bile acid metabolism such as *Anaerotruncus colihominis*, *Intestinibacillus sp*. Marseille-P4005, *Oscillibacter sp*. PEA192, *Flavonifractor plautii* and *Mediterraneibacter glycyrrhizinilyticus*, and two *Lachnospiraceae* species previously linked to bile acid metabolism: *Enterocloster citroniae* and *Blautia obeum*. Collectively, our results indicate that certain related species are associated with multiple metabolites within the same class of metabolites; this was especially prominent for secondary bile acids and their precursors.

### Functions shared by several species are linked to single metabolite abundance

Different microbiota species may share genetic elements that enable them to perform the same metabolic function. We hypothesized that such genetic elements shared by several species affect single metabolite levels. We therefore mapped microbial genes to 103 gut metabolic modules (GMM), in order to associate microbial metabolic function with species associated with single metabolites. In total, we found an enrichment of 90 unique microbial functions for 1295 plasma metabolites (18,339 total enrichments, *q*-value < 0.05, Supplementary Data 9). Among the microbial functions with strongest enrichments, those functions encoding enzymes catalyzing the degradation of amino acids and monosaccharides such as threonine (e.g. with gentisate), serine (e.g. with 1-(1-enyl-palmitoyl)−2-arachidonoyl-GPE (P-16:0/20:4)), sucrose (e.g. with chenodeoxycholate), ribose (e.g. with tyramine O-sulfate), and fructose (e.g. with pantothenate) were most prominent. Overall, these findings support that certain functions shared by several species are common in species that are associated with plasma metabolite abundances.

The above analyses clearly show the existence of specific associations between gut microbiota and the host plasma metabolome. Below, we present detailed data of the association of selected microbes and metabolites as case examples of information that can be mined using the GUTSY Atlas of associations of the plasma metabolome with gut microbiota. We focused on microbiota associations with the uremic toxin *p*-cresol sulfate, as an example of a bacteria-derived metabolite implicated in human health; and with omeprazole and metformin, which are common medications that are thought to have profound effects on the microbiota; and with coffee metabolites, as an example of a common dietary component reported to have large effects on the microbiota. A summary of all the results for these metabolites can be found in Supplementary Data 10.

### *Faecalibacterium prausnitzii* and *Intestinimonas massiliensis* are strongly associated with the uremic toxin *p*-cresol and phenylacetylglutamine, but in opposite directions

In the current study, we observed that 36% of the variation in *p*-cresol sulfate plasma levels was explained by the variation in gut microbiota — one of the highest proportions of explained variation of all metabolites. The bacterial metabolite *p*-cresol is classified as a uremic toxin and is produced during bacterial tyrosine fermentation in the large intestine and accumulated in patients with kidney failure, and its levels are associated with worse outcomes^24,25^. In a germ-free mouse model of chronic kidney disease, transplantation of fresh microbiota from end-stage renal disease patients led to increased serum levels of *p*-cresol sulfate and other uremic toxins compared to serum samples from mice transplanted with microbiota from healthy donors^26^. This was interpreted to mean that the aberrant gut microbiota in renal patients aggravates the disease by modulating uremic toxin levels, and highlights the importance of better characterization of the uremic toxin-producing microbiota. In our data, lower eGFR was associated with higher levels of 10 established and proposed uremic toxins (Spearman’s *p*-value < 10^−12^, Supplementary Data 11). We found that *p*-cresol sulfate and the related metabolite *p*-cresol glucuronide as well as the glutamine-derived phenylacetylglutamine^27^ showed much stronger associations with several species from the Eubacteriales order (*p*-values = <10^−200^), including novel positive associations with *Intestinimonas massiliensis*, than other established and proposed uremic toxins, such as hippurate, indoxyl sulfate, TMAO, and 3-carboxy-4-methyl-5-propyl-2-furanpropanoic acid (Supplementary Fig. 2). This association supports that members of the Eubacteriales order, formerly called Clostridiales, is one of the most prolific phenol compound-generating bacterial subgroups that produce *p*-cresol sulfate as a tyrosine fermentation end product^28^. Importantly, we also found several strains of *Faecalibacterium prausnitzii* strongly inversely associated with *p-*cresol levels and phenylacetylglutamine. Interestingly, *F. prausnitzii* was one of the depleted species in the microbiota of renal patients, compared to healthy controls^26^, and its reduced levels have been linked to more severe stages of renal disease^29^. We performed additional models stratified by eGFR, and found slightly stronger associations in the individuals with lower kidney function (Supplementary Fig. 2). In summary, we identify a number of species that are strongly positively or negatively associated with *p*-cresol and phenylacetylglutamine levels, which sets the foundation for future studies into perturbation of the gut flora to reduce uremic toxins.

### Increased abundance of oral bacteria and enrichment of functions related to carbohydrate metabolism in omeprazole users

Omeprazole is a selective proton pump inhibitor (PPI) commonly used for treatment of acid-related upper gastroduodenal diseases, and sold over-the-counter as well as by prescription. We compared dispensed omeprazole prescriptions with the plasma levels of omeprazole, and observed that out of the 329 (4%) participants with detectable plasma levels of omeprazole, 67% had a dispensed prescription for omeprazole in the past 12 months, while for the 8254 participants with non-detectable levels, 8% had a prescription (Fisher’s exact test *p*-value = 4.0 × 10^−^^147^). In the present study, we observed strong positive associations between presence of omeprazole in plasma and bacteria belonging to the *Veillonella* genus (e.g., *V. parvula*, *V. dispar* and *V. atypica*) and *Streptococcus* genus (e.g., *S. anginosus*, *S. oralis subsp oralis*, *S gordonii*, *S. salivarius*, *S. parasanguinis* and *S. mutans*), all parts of the normal oral microbiota. This expands previous findings from a recent studies^13,30^ that reported that PPI use was associated with an increased abundance of several taxa common to the oral flora, such as *Veillonella* and several *Streptococcus* species. Interestingly, *V. parvula* is reported to have a mutualistic relationship with *S. mutans* by co-aggregating and transforming metabolic products of other carbohydrate-fermenting bacteria^31^. With regards to the potential function of omeprazole-associated bacteria, we found that functional modules linked to degradation of fructose, ribose, lactate and trehalose were strongly enriched (all *p*-values <10^−6^) for bacterial species positively associated with omeprazole, pointing again to carbohydrate-fermentation. Although we only investigated omeprazole and no other types of PPI, earlier studies have demonstrated similar effects of different PPI types on the gut microbiota^13,30^. Taken together, the current study provides strong support for the notion that PPI use is associated with consistent alteration of gut microbiota, characterized by the increased abundance of bacteria common in the oral flora with an enrichment for bacterial functions related to carbohydrate metabolism.

### Profound changes of the gut microbiota composition and high abundance of bacteria carrying genes enabling amino acid metabolism with metformin treatment

Metformin is a widely used anti-diabetic drug that has been associated with profound changes in the gut microbiota composition, and also with gastrointestinal side effects such as bloating and discomfort^32,33^. We compared dispensed metformin prescriptions with the plasma levels of metformin (metformin is not metabolized in the body), and observed that out of the 371 (4%) participants with detectable plasma levels of metformin, 78% had a dispensed prescription for metformin in the past 12 months, while for the 8212 participants with non-detectable levels, only 0.2% had a prescription (Fisher’s exact test *p*-value = <10^−300^). Here we identified 462 species, whose abundances were associated with plasma metformin, of which an increased abundance of *Escherichia marmotae* and *E. coli*, and decreased abundance of *Romboutsia timonensis*, *Intestinibacter sp*. MGS:0194 and *Intestinibacter bartlettii* were the strongest associations. These top findings are in accordance with earlier studies reporting a significant enrichment of *E. coli* in the gut microbiota of metformin users^13,32,33^ and a decreased abundance in *R. timonensis* and *I. bartletii*^13^, as well as with a recent randomized trial that showed that metformin treatment in overweight/obese individuals results in an increased abundance of *E. coli* and a decreased abundance of *I. bartlettii* at 6 and 12 months of metformin treatment^34^. Further, an increase of *Ruminococcus torques* was reported at both time points in that study^34^, which is also supported by an earlier study^13^ and our study (*p*-value = 3.8 × 10^−10^). *R. timonensis* is a new species that was recently isolated from the human gut^35^ and has not been associated with use of metformin prior to the Mueller et al. study^34^. In species associated with metformin, we found strong positive enrichments for bacterial functional modules involved in amino acid metabolism, namely the degradation of isoleucine and alanine, which are previously reported to increase during metformin treatment^36,37^. In addition, we found functional modules involved in carbohydrate metabolism, such as the degradation of fructose and trehalose, in line with data from an intervention study^33^. Taken together, our results confirm and expand previous findings that metformin treatment is associated with profound changes of the gut microbiota composition, and that bacteria carrying genes enabling amino acid and carbohydrate metabolism are in higher abundance in metformin users.

### Coffee metabolites have strong positive associations with species from the Eubacteriales order

Coffee is one of the most widely consumed beverages in the world and has a complex and not fully elucidated relationship with human health^38^. The PREDICT1 (*n* = 1098) study revealed a large number of diet-microbiota associations, of which the strongest combined associations were found for coffee intake^12^. We sought to further investigate the links between microbiota characteristics and coffee using the GUTSY Atlas data by investigating 12 established coffee metabolomic biomarkers^39, 40^. In our data, higher levels of these 12 coffee metabolites were all associated with higher self-reported coffee intake in a dose-dependent manner (Spearman’s *p*-value < 10^−24^, Supplementary Data 12). We observed that 21 individual species in different combinations represented the eight most strongly associated species for each of these 12 biomarkers (as depicted in Supplementary Fig. 3). These 21 species were all from the Eubacteriales order from the *Ruminococcaceae*, *Oscillospiraceae*, *Lachnospiraceae* and *Clostridiaceae* families, except *S. salivarius*. Three species were annotated at the species level: *C. phoceensis*, *Anaeromassilibacillus sp*. Marseille-P3371 and *S. salivarius*, which were all associated in the positive direction with all the 12 coffee biomarkers. *C. phoeensis* was first identified in the gut microbiota of a healthy 28-year-old man in Marseille^41^ and has not previously been linked to any phenotypes. *Anaeromassilibacillus sp*. Marseille-P3371 has been found to be affected by a low-protein diet in a dietary trial of chronic kidney disease patients. The commensal bacterium *Streptococcus salivarius* is one of the early bacteria colonizing the oral and gut mucosal surfaces. This species is proposed to have positive effects in the oral cavity and upper respiratory tract; it may inhibit colonization of other pathogens such as *S. pyogenes*^42^ and virulent *Streptococcus* species involved in tooth decay such as *S. mutans*^43^, and also has anti-inflammatory characteristics. It is currently not known why the abundance of certain gut bacteria is positively associated with coffee intake. It does not seem to be driven by smoking behavior (Supplementary Fig. 3), but it may be related to the metabolism of these bacteria. Of note, coffee is rich in antioxidants^44^ and affects gut motility, which could also affect the sampling and the bacterial community^45^. In summary, we report novel association of previously reported coffee biomarkers with the abundance in the gut microflora with a set of bacteria from the Eubacteriales order and with *S. salivarius*.

## Discussion

We performed the largest and most detailed association study of the gut microbiota and host plasma metabolites to date and present the results as the online GUTSY Atlas, which can be used as the starting point for targeted studies of perturbation of specific microbial species and to identify candidate plasma biomarkers of gut flora composition. The analysis revealed 546,819 associations of individual microbial species with metabolites, and confirmed and substantially expanded previous studies in the area^6–12^. This resource is non-targeted and therefore encompasses large parts of the described and undescribed human gut microbial community and the plasma metabolome, enabling researchers with varying interests to benefit from the data.

We observe a large variation in the association of gut microbiota species with plasma metabolites, where certain metabolites such as *p*-cresol and secondary bile acids have strong associations with multiple bacterial species, and others, such as nucleotides, have few associations. We report that certain species are associated with multiple metabolites within the same class of metabolites; this was especially prominent for secondary bile acids and their precursors, secondary bile acids are produced by the gut bacteria and involved in fat and oil digestion^46^.

We also detect a number of novel observations in terms of specific biomarkers, metabolites and drugs, such as the association of coffee biomarkers with a set of bacteria from the Eubacteriales order and with *S. salivarius*, which is regarded as a competitor to more pathogenic strains of the genus *Streptococcus*. These results support previous findings that coffee intake, one of the most consumed beverages globally, has effects on the composition of the gut microbiota, which warrants further investigations in the possible links with health.

We find a number of species strongly associated with *p*-cresol levels. *P*-cresol is regarded as an important uremic toxin, produced during bacterial tyrosine fermentation in the large intestine and accumulated in patients with kidney failure. This causes further damage to the kidney, and can only be marginally removed by dialysis^24, 25^. We identify several substrains of *F. prausnitzii* with variable association with *p*-cresol, indicating that certain strains may have larger effects than others. The results from the GUTSY Atlas could hence be used as a foundation for designing future studies of gut flora perturbation in chronic kidney failure to reduce uremic toxins with the purpose to decrease kidney disease progression.

Our study provides strong support for the notion that PPI use is associated with consistent alteration of gut microbiota, characterized by the increased abundance of bacteria common in the oral flora with an enrichment for bacterial functions related to carbohydrate metabolism. To find oral species in the gut microbiota is not uncommon. Recent research has shown that about 40% of the common species (≥10% prevalence) are shared between the oral and gut microbiota communities, although the relative abundance differed greatly between these two sites^47^. PPI use is common in the population and found associated with a number of traits in observational studies, such as small intestine bacterial overgrowth. The health impact of PPI-related changes of the gut microbiota warrants further investigation.

Our findings also confirm many of those reported in the previously largest high-resolution gut microbiome – plasma metabolome study from the TwinsUK adult twin registry (*n* = 859)^8^. For example, for the top 10 associations annotated to the species level in the TwinsUK, nine were available in our study of which seven were replicated, i.e., associations between *F. prausnitzii* and *p*-cresol sulfate, *p*-cresol glucuronide, phenylacetylglutamine and deoxycholate, *Methanobrevibacter smithii* and threonate, *Roseburia inulinivorans* and *p*-cresol sulfate, and *E. coli* and phenylacetylglutamine. Interestingly, their top finding of a strong association of *Barnesiella intestinihominis* with plasma levels of sebacate (decanedioate) was not replicated in our study, although both the species and the metabolite were present in our data. Further, in the current study, the gut microbiota explained 46% of the variance in the uncharacterized molecule X-11850 and explained >25% of the variance in other metabolites, such as coffee metabolite quinate (*r*^2^ = 0.27) and uremic toxin *p*-cresol sulfate (*r*^2^ = 0.36). This aligns with the recent study by Bar et al.^10^, which analyzed these associations using similar methods but in two smaller Israeli cohorts (*n* = 491, replication in 1004 participants from TwinsUK and 245 from IMI-DIRECT)^10^. For example, in the study by Bar et al., X-11850 exhibited the second highest variance explained (*r*^2^ = 0.49), and quinate (*r*^2^ = 0.45) and *p*-cresol sulfate (*r*^2^ = 0.41) were also among the top 10 highest variance explained. The identified large overlaps with previous studies and the current study indicates that findings are in general robust over different populations and analytical platforms. Given the improved statistical power in the current study, we expanded the number of findings from 254 associations of the TwinsUK study to 546,819 associations in the GUTSY Atlas, also including associations of moderate effect size and for more rare species. We also confirm and expand previous findings that metformin treatment is associated with profound changes of the gut microbiota composition, and that bacteria carrying genes enabling carbohydrate metabolism are in higher abundance in metformin users, in line with data from an intervention study^33^.

### Strengths and limitations

The major strengths of the current study are the sample size, high-resolution data and the easy-to-use companion website. While we replicated the findings of other studies, confirming the quality of the generated data, we also identified many novel associations between oral medication and the gut microbiome, and microbiota species strongly associated with levels of the uremic toxin *p*-cresol sulfate. The cohort analyzed in the current study is more than three times bigger than the previously largest study in which gut microbiota were analyzed by 16S sequencing and associated with NMR-based plasma metabolome profiling (*n* = 2309)^7^ and 10 times bigger than that of a previously largest study in which gut microbiota were analyzed by high-resolution metagenomics and associated with mass spectrometry-based plasma metabolome profiling (*n* = 859)^8^, which allowed us to also assess associations of moderate size and with rare metabolites and species. Another strength is the deep phenotyping of the SCAPIS study, which allowed detailed sensitivity analyses of potential confounders and effect modifiers^14^. We also observed strong concordance between lower kidney function and higher uremic toxin levels, between dispensed prescriptions and plasma levels of drugs, and between self-reported coffee intake and plasma coffee metabolite levels. Consequently, the presented association atlas is based on a large well-characterized sample and state-of-the art analytical methods for microbiota and metabolomics which will enable well-powered in silico exploration of the potential metabolic effect of various bacteria of interest and for identifying candidate plasma biomarkers of gut flora composition.

However, some limitations of the present study should be recognized. First, the study population comprises mostly Scandinavian-born participants aged 50﻿–65 of predominantly European descent. While the top findings for this cohort were similar to those of samples for the UK (mean age: 65 years) and Israel (age: 18–70 years), generalizations for other species-metabolites associations to other populations and age groups need further investigation. Second, the observational nature of the cross-sectional study design makes residual confounding a potential issue and causal inference difficult. Nonetheless, for food-derived metabolites, such as the coffee metabolite quinate, and drugs, such as omeprazole and metformin, the causal direction from the medication/food intake to the gut microbiota is most likely, although it could still be confounded by factors that co-vary with the food and medication type. Conversely, for metabolites produced by the gut microbiota, such as secondary bile acids, we assume the causal direction from the gut microbiota to the plasma metabolome. Any causal links should, however, be verified in the future by using experiments or causal inference methods, such as Mendelian randomization^48,49^. Third, similar to previous studies, the associations were analyzed using rank-based non-parametric models, which hinders the interpretation of actual effect sizes. Fourth, due to the compositionality of the microbial species data, there might be an inflated number of false positives in our main model. We therefore also report the results of a sensitivity analysis additionally adjusted for alpha diversity, which is potentially less susceptible to this issue, at the cost of increased risk of bias and lower power. Metagenomic sequencing methods that can accurately quantify the actual levels of microbial species will be required to fully solve this issue. Fifth, although the number of annotated metabolites and species is high in this study, many of the identified associations were between unknown metabolites and species for which no reference genome is currently available, which makes the interpretation of some of the novel findings in context challenging. We plan to update the companion website as additional metabolites and species are characterized in the future.

In summary, we here identified a vast number of robust associations between the gut microbiota and the plasma metabolome, and report these to the community in the GUTSY Atlas, a comprehensive online resource for an interactive investigation of the associations. These findings add to the knowledge of the vast interactions of the gut microbiota and human metabolism and will generate insights into human biology and identification of potential novel biomarkers of gut flora composition. We anticipate that the GUTSY Atlas will be of immense benefit for the scientific community, reducing the need for collecting and analyzing their own samples.

## Methods

### Study sample

SCAPIS is a prospective population-based study of 30,154 men and women, aged 50–65 years, living in six municipality regions in Sweden. It was designed with the main aim to improve risk prediction and understanding of cardiovascular disease, chronic pulmonary obstructive disease, and related metabolic disorders^14^. After a pilot study in 2012, recruitment was initiated in 2014 and completed in 2018. Individuals were randomly recruited from the population register, with a participation rate of 50%.

The present study is based on a subset of the 11,287 participants from Uppsala (*n* = 5036) and Malmö study sites (*n* = 6251). Participants at both centers followed a 3-day-visit scheme, where anthropometric measurements, dietary questionnaire, and blood draw were performed at visit 1 and blood pressure measurements at visit 2. Fecal sampling and health and lifestyle questionnaires were completed at home between visits 1 and 2. Once collected, fecal samples were stored at −20 °C until visit 2. The average number of days between visits 1 and 2 were 15 days for Uppsala and 11 days for Malmö.

Of these 11,287 participants, 9831 had their fecal samples processed for metagenomics analysis. However, 12 samples were excluded as they provided a low DNA yield and/or had features interfering with the library preparation even after one attempt of re-extraction each. Furthermore, one sample with only 1473 reads mapped to the signature genes was removed after sequencing. Hence, 9818 participants were deemed to have high-quality fecal metagenomics data (Uppsala, *n* = 4838; Malmö, *n* = 4980).

Out of the 8962 SCAPIS plasma samples processed for metabolome profiling, two samples were lost during processing, two samples were excluded whose data were determined to be outliers based on principal component analysis, and one sample was excluded based on clearly discordant levels of glucose, cholesterol, and creatinine compared to the reference laboratory measurement. Hence, 8957 participants (Uppsala, *n* = 4979; Malmö, *n* = 3978) were deemed to have high-quality plasma metabolomics data. The metagenomics and metabolomics data were then combined, yielding an overlapping dataset of 8616 participants (Uppsala, *n* = 4787; Malmö, *n* = 3829). We further excluded 33 participants (Uppsala, *n* = 15; Malmö, *n* = 18) with missing data for covariates in the main model, yielding a final study sample of 8583 participants (Uppsala, *n* = 4772; Malmö, *n* = 3811) in the present study.

All study participants provided a signed informed consent at the first site visit. The study adheres to the Declaration of Helsinki and was approved by the Swedish Ethics Review Authority (Etikprövningsmyndigheten Dnr 2010-228-31M, Dnr 2018-315). The participants received no compensation for their participation.

### Gut microbiome sample collection and preprocessing

At the first visit, participants received a pre-packaged fecal sample collection kit (barcoded tubes, gloves, Ziploc bags, and a paper collection bowl) including instructions on how to collect the sample at home. The participants were asked to store the samples at –20 °C in the home freezer until the study site visit. The participants were asked to store the samples at −20 °C in the home freezer until the study site visit. Of the 8538 samples that were part of this study, 8131 samples were returned at visit 2 (Uppsala, *n* = 4629; Malmö, *n* = 3502), 221 (Uppsala, *n* = 107; Malmö, *n* = 114) samples were received within a week after visit 2, and 139 (Uppsala, *n* = 31; Malmö, *n* = 108) 8 days or later after visit 2. Once received in the laboratory, the samples were kept at –20 °C for 0–7 days until transport to the central biobank for storage at –80 °C. Finally, the samples were shipped on dry ice to Clinical Microbiomics A/S (Copenhagen, Denmark) for metagenomics analysis. Samples were analyzed in a random order and 158 samples were analyzed in replicate.

Fecal DNA was extracted using NucleoSpin® 96 Soil kit (740787; Macherey-Nagel; Germany). Negative and positive controls were included. Samples were subjected to 5 min of bead beating at 2200 rpm, with 1.2 μg of DNA obtained on average per sample. The sequencing of metagenomes was performed using 2 × 150 bp paired-end sequencing on Illumina Novaseq 6000 system (Illumina, USA). On average, 26.3 million read pairs (7.9 Gb) were generated per sample for Malmö samples, and 25.3 million read pairs (7.6 Gbp) were generated per sample for Uppsala samples. Sequencing reads with adapters, containing >10% ambiguous bases, those with >50% bases with Phred quality score <5, and reads mapped to the human reference genome GRCh38 were removed using Bowtie 2 v02.3.2^50^ with default settings. The remaining reads (median: 24.8 × 106, minimum: 8.2 × 106 read pairs per sample) were assembled using MEGAHIT v1.1.1^51^ and mapped using BWA mem v0.7.16a^52^ to a newly created gene catalog of 14 million non-redundant microbial genes from three main sources: data from the present study and the Malmö offspring study^53^, data from Pasolli et al.^54^, and 3488 publicly available genomes of isolated microbial strains relevant to the human gut microbiome^55–57^. Metagenomic species were defined as co-abundant gene groups from the gene catalog that fulfilled previously established quality criteria^58^. In each species, 100 highly correlated and distinct signature genes were identified and used for abundance profiling. Overall, 1985 species were identified in the gene catalog. The number of gene counts for every sample mapped to the signature genes of a species determined the count number of that species. However, the count number was set to zero for any species with reads mapping to <3 of its signature genes. Species relative abundances were calculated by dividing the counts of each species by the effective length of its signature genes, and then normalizing each sample to sum to 100%. The species relative abundances were (natural) log+1 transformed, and species with at least 100 non-zero measurements were included in further analyses. After quality control, 1528 species were included, with an average of 325 per sample. Shannon diversity was calculated using the *vegan*^59^ v2.5-7 R package on downsized data to avoid confounding by read depth. The reads in each sample were downsized by random sampling without replacement from the gene count table corresponding to the signature genes to 210,430 reads. One sample with only 1473 reads mapped to the signature genes was discarded. For taxonomic annotation, catalog genes were compared to those in the NCBI RefSeq^60^ database (https://www.ncbi.nlm.nih.gov/refseq/, downloaded on 2 May 2021). Species-level taxonomy was assigned to metagenomic species with ≥75% of genes with ≥95% sequence similarity to a single species. For the genus, family, order, class, and phylum annotations, different thresholds were used (≥60, 50, 40, 30, and 25% of genes; with ≥85, 75, 65, 55, and 50% sequence similarity, respectively).

For functional annotation, catalog genes were annotated to the gut metabolic modules v1.07^61^ (https://github.com/raeslab/GMMs, GMM) database using EggNOG-mapper^62^ v2.0.1. Potential functional profiles were determined for species that contained at least 2/3 of the enzymes/protein genes needed for the functionality of a particular GMM module. If an alternative reaction pathway within a module existed, only one such reaction pathway was required. All reaction pathways were required for modules with fewer than four steps.

### Plasma metabolome sample collection and preprocessing

Venous blood samples were collected from the participants during the study site visit after an overnight fast. The samples were stored at –80 °C in the biobank until shipping to Metabolon Inc. for plasma metabolome analysis (Durham, NC, USA)^63^. Samples were handled and analyzed in random order together with different quality control standards, namely, pure water, solvents used for metabolite extraction, a pool of human plasma samples maintained by Metabolon Inc., and a pool of study participants’ samples. Proteins were removed by methanol precipitation with vigorous shaking using Glen Mills GenoGrinder 2000 and centrifugation. To maximize metabolite identification, four processes were used in parallel: reverse phase (RP)/ultrahigh performance liquid chromatography–tandem mass spectroscopy (UPLC-MS/MS) with negative-ion mode electrospray ionization (ESI), hydrophilic interaction chromatography (HILIC)/UPLC-MS/MS, and two separate RP/UPLC-MS/MS resolutions with positive-ion mode ESI. Peak identification and quantification, and quality control were performed using Metabolon’s hardware and software. For each metabolite, for each instrument plate (144 samples), the peak measurement areas were divided by the median peak area of samples in that batch. Metabolite measurements that failed to reach the detection threshold were imputed from the minimum observed value for that metabolite. Metabolites were annotated by matching to Metabolon’s library of more than 3300 purified standards and unknown compounds based on the RI, m/z, and chromatography data. As part of the annotation process, two types of metabolic pathways were assigned to each metabolite: (1) “metabolite class”, which includes broad metabolic pathway terms, and (2) “metabolite subclass”, which includes narrow metabolic pathway terms. Metabolites with at least 100 measurements above the detection threshold were included in the present study. Metabolites other than drug metabolites were (natural) log+1 transformed. Metabolites classified as drugs in the xenobiotics class by Metabolon were converted to binary values (present or absent). Overall, 1321 metabolites that passed quality control were included in the analyses. Of those, 269 metabolites were not annotated, 238 metabolites were not confirmed with an internal standard, 142 metabolites were not measured in at least one of the 3 metabolomics delivery batches, and 76 metabolites were only measured for 8582 of the 8583 participants, since one of the samples was lost during processing for the hydrophilic interaction chromatography (HILIC)/UPLC-MS/MS measurement.

### Phenotype processing

Self-reported coffee intake was collapsed from a total of 8 categories to 4 categories (<1, 1-2, 3-4, and >4 cups per day). Place of birth was determined by collapsing self-reported country of birth into categories Scandinavia, non-Scandinavian Europe, Asia, or other. Metformin (ATC codes A10BA02, A10BD20, A10BD07, A10BD05, and A10BD03), omeprazol (ATC code A02BC01), and antibacterials for systemic use (ATC codes J01) dispensed prescriptions were retrieved from the Swedish Prescribed Drug Register using the period from 12 months prior to visit 1 in SCAPIS. Estimated glomerular filtration rate was calculated with the CKD-EPI Study equation^21^. To adjust for total energy intake, fiber intake was divided by total energy intake.

### Statistical analysis

Analyses were performed and plots were created with R v4.1.1 (https://cran.r-project.org/). Partial Spearman’s rank correlations were calculated for Shannon diversity index and each metabolite using the *ppcor*^64^ v1.1 R package. Correlation estimates were adjusted for age, sex, place of birth (Scandinavia, non-Scandinavian Europe, Asia, or other), study site (Uppsala or Malmö), microbial DNA extraction plate, and metabolomics delivery batch, which denotes three batches of samples (two from Uppsala and one from Malmö) that were profiled separately, and later normalized jointly. Microbial extraction plate and metabolomics delivery batch were nested in study site, and therefore study site, microbial extraction plate and metabolomics delivery batch were combined into one categorical batch variable by combining the labels. The categorical variables with more than two levels (place of birth and batch) were converted to dummy variables and the dummy variable of the last category was removed before analysis. Association *p*-values were adjusted for multiple testing using the Benjamini–Hochberg method at 5% false discovery rate. Similarly, for each species, partial Spearman’s rank correlations were calculated for each metabolite and adjusted for age, sex, place of birth, study site (Uppsala or Malmö), microbial DNA extraction plate, and metabolomics delivery batch as the main model, and additionally adjusted for Shannon diversity index as a sensitivity analysis. Further sensitivity analyses were performed for the main model by stratification of the participants by tertiles of body mass index, systolic blood pressure, estimated glomerular filtration rate, fiber intake, and by exclusion of smokers and participants who had been prescribed antibiotics within a year of visit 1 or taken, according to the health and lifestyle questionnaire, medication for hypertension, dyslipidemia, and/or diabetes in the last two weeks.

Ridge regression models were used to estimate the proportion of variance of each metabolite explained by gut microbiota based on a nested 10-fold cross-validation approach using the *glmnet*^65^ v4.1-3 R package. For each iteration, the data was split into 10 folds. Nine of the folds were used as training folds and one as a hold-out fold. The 9 training folds were in turn split into 10 additional folds, and a grid search was performed to find the lambda corresponding to the lowest cross-validated mean squared error. The performance of the model with this lambda was then tested on the hold-out fold. This procedure was performed 10 times, each time changing the hold-out fold, and the average training mean squared error, the average test mean squared error and the average test *r*^2^ were reported.

For 50 uncharacterized metabolites found to have highest variance explained by the microbiome, additional searches for the two most common ion adducts (M + H and M + Na for positive, M-H and M-Cl for negative mode) with a 5 parts per million tolerance for difference in m/z in METLIN^66^ were performed. This search did not yield any additional conclusive annotations, as there were several different matches for each m/z value.

Enrichment analysis of ranked association *p*-values (ties broken by absolute *t*-statistic) was performed using the *fgsea*^67^ v1.19.2 package for positive and negative Spearman’s *ρ* separately as one-sided tests. The enrichment *p*-values for positive and negative coefficients were combined and adjusted for multiple testing using the Benjamini–Hochberg method at a 5% false discovery rate. Enrichment analysis was done using GMM modules and metabolite subclasses, with a minimum of 5 metabolites or species per group, respectively.

### Reporting summary

Further information on research design is available in the Nature Research Reporting Summary linked to this article.

## Supplementary information

Supplementary Information

Description of Additional Supplementary Files

Supplementary Data 1

Supplementary Data 2

Supplementary Data 3

Supplementary Data 4

Supplementary Data 5

Supplementary Data 6

Supplementary Data 7

Supplementary Data 8

Supplementary Data 9

Supplementary Data 10

Supplementary Data 11

Supplementary Data 12

Reporting Summary

## Data Availability

De-hosted anonymized metagenomic sequencing data generated in this study have been deposited in the European Nucleotide Archive under accession number PRJEB51353 (https://www.ebi.ac.uk/ena/browser/view/prjeb51353). Metabolomics analysis was performed at Metabolon, TX, USA, who deposited spectral data from the first analytical stage (MS1) for 125 anonymized samples from SCAPIS-Uppsala in MetaboLights under accession number MTBLS407. However, MS/MS spectral data are not shared by Metabolon to the research community. Additional individual-level data are available under restricted access as they contain sensitive personal information that are protected under privacy laws, and access can be obtained following ethical approval from the Swedish Ethical Review Board (https://etikprovningsmyndigheten.se/; the application procedure and instructions are provided in the link) and data access approval from the SCAPIS Data access board (https://www.scapis.org/data-access/; the application procedure and conditions are provided in the link). These data may only be used for research, and are not available for commercial use. Underlying data for all figures are provided in the Source Data file and available at https://github.com/MolEpicUU/GUTSY_Atlas^68^. A companion website to the article containing the full results of the current study and further study-related searchable material can be accessed via https://gutsyatlas.serve.scilifelab.se/.

## Code availability

Code related to the analyses in this study are available at https://github.com/MolEpicUU/GUTSY_Atlas.
