# Author Correction: An online atlas of human plasma metabolite signatures of gut microbiome composition

**DOI:** 10.1038/s41467-023-38607-1

**Published:** 2023-05-23

**Authors:** Koen F. Dekkers, Sergi Sayols-Baixeras, Gabriel Baldanzi, Christoph Nowak, Ulf Hammar, Diem Nguyen, Georgios Varotsis, Louise Brunkwall, Nynne Nielsen, Aron C. Eklund, Jacob Bak Holm, H. Bjørn Nielsen, Filip Ottosson, Yi-Ting Lin, Shafqat Ahmad, Lars Lind, Johan Sundström, Gunnar Engström, J. Gustav Smith, Johan Ärnlöv, Marju Orho-Melander, Tove Fall

**Affiliations:** 1 Department of Medical Sciences, Molecular Epidemiology and Science for Life Laboratory, Uppsala University, Uppsala, Sweden; 2 CIBER Cardiovascular diseases (CIBERCV), Instituto de Salud Carlos III, Madrid, Spain; 3 Division of Family Medicine and Primary Care, Department of Neurobiology, Care Science and Society, Karolinska Institute, Huddinge, Sweden; 4 Department of Clinical Sciences, Lund University, Malmö, Sweden; 5 Clinical Microbiomics A/S, Copenhagen, Denmark; 6 Department of Medical Sciences, Uppsala University, Uppsala, Sweden; 7 Department of Medical Sciences, Clinical Epidemiology, Uppsala University, Uppsala, Sweden; 8 The George Institute for Global Health, University of New South Wales, Sydney, Australia; 9 The Wallenberg Laboratory/Department of Molecular and Clinical Medicine, Institute of Medicine, Gothenburg University and the Department of Cardiology, Sahlgrenska University Hospital, Gothenburg, Sweden; 10 Department of Cardiology, Clinical Sciences, Lund University and Skåne University Hospital, Lund, Sweden; 11 Wallenberg Center for Molecular Medicine and Lund University Diabetes Center, Lund University, Lund, Sweden; 12 School of Health and Social Studies, Dalarna University, Falun, Sweden

**Keywords:** Metabolomics, Microbial genetics

Correction to: *Nature Communications* 10.1038/s41467-022-33050-0, published online 23 September 2022

The original version of this Article contained errors related to reporting of the estimation of the variance explained as follows:

1. The original version of the Abstract incorrectly read ‘Here, we find that the gut microbiota explains up to 58% of the variance of individual plasma metabolites and we present 997 associations between alpha diversity and plasma metabolites and 546,819 associations between specific gut metagenomic species and plasma metabolites in an online atlas (https://gutsyatlas.serve.scilifelab.se/).’ The correct version replaces this sentence with ‘Here, we find that the gut microbiota explains up to 46% of the variance of individual plasma metabolites and we present 997 associations between alpha diversity and plasma metabolites and 546,819 associations between specific gut metagenomic species and plasma metabolites in an online atlas (https://gutsyatlas.serve.scilifelab.se/).’
2. The original version of this Article contained an error in the Results, which incorrectly read ‘We observed that the variance of 1179 of the 1321 metabolites was partly explained by variation in the gut microbiota (Fig. 2a and Supplementary Data 5). We detected the largest variance explained (58%) for an uncharacterized common metabolite with the provisional identifier X-11850. The main feature mass-to-charge ratio (m/z), retention-time index (RI), and measurement platform for all uncharacterized and characterized metabolites are reported in Supplementary Data 2. However, MS/MS spectral data are not shared by the external laboratory (See Data Availability Statement). The variance explained by gut microbiota species was >15% for 554 metabolites and >30% for 61 metabolites, such as uremic toxin p-cresol sulfate (*r*^*2*^ = 46%) and the coffee metabolite quinate (*r*^*2*^ = 45%). For trimethylamine N-oxide, TMAO, the end-product of diet-microbiota interaction, which has been suggested involved in cardiovascular and kidney disease pathogenesis20, we found a rather low variance explained by the gut microbiota (*r*^*2*^ = 12%).’ The correct version replaces these sentences with ‘We observed that the variance of 1168 of the 1321 metabolites was partly explained by variation in the gut microbiota (Fig. 2a and Supplementary Data 5). We detected the largest variance explained (46%) for an uncharacterized common metabolite with the provisional identifier X-11850. The main feature mass-to-charge ratio (m/z), retention-time index (RI), and measurement platform for all uncharacterized and characterized metabolites are reported in Supplementary Data 2. However, MS/MS spectral data are not shared by the external laboratory (See Data Availability Statement). The variance explained by gut microbiota species was >10% for 133 metabolites and >25% for 22 metabolites, such as uremic toxin p-cresol sulfate (*r*^*2*^ = 36%) and the coffee metabolite quinate (*r*^*2*^ = 27%). For trimethylamine N-oxide, TMAO, the end-product of diet-microbiota interaction, which has been suggested involved in cardiovascular and kidney disease pathogenesis20, we found a rather low variance explained by the gut microbiota (*r*^*2*^ = 1.7%).’
3. The original version of this Article contained an error in the Results, which incorrectly read ‘Both the primary bile acid cholic acid (denoted cholate in the atlas) and the secondary bile acid deoxycholic acid (deoxycholate) had a high variance explained by the microbiota (*r*^*2*^ = 30% and 36%, respectively), indicating a strong impact of the variation in microbiota composition.’ The correct version replaces this sentence with ‘Both the primary bile acid cholic acid (denoted cholate in the atlas) and the secondary bile acid deoxycholic acid (deoxycholate) had a high variance explained by the microbiota (*r*^*2*^ = 18% and 22%, respectively), indicating a strong impact of the variation in microbiota composition.’
4. The original version of this Article contained an error in the Results, which incorrectly read ‘In the current study, we observed that 46% of the variation in *p*-cresol sulfate plasma levels was explained by the variation in gut microbiota—one of the highest proportions of explained variation of all metabolites.’ The correct version replaces this sentence with ‘In the current study, we observed that 36% of the variation in *p*-cresol sulfate plasma levels was explained by the variation in gut microbiota—one of the highest proportions of explained variation of all metabolites.’
5. The original version of this Article contained an error in the Discussion, which incorrectly read ‘Further, in the current study, the gut microbiota explained 58% of the variance in the uncharacterized molecule X-11850 and explained >30% of the variance in other metabolites, such as coffee metabolite quinate (*r*^*2*^ = 0.45) and uremic toxin p-cresol sulfate (*r*^*2*^ = 0.46).’ The correct version replaces this sentence with ‘Further, in the current study, the gut microbiota explained 46% of the variance in the uncharacterized molecule X-11850 and explained >25% of the variance in other metabolites, such as coffee metabolite quinate (*r*^*2*^ = 0.27) and uremic toxin p-cresol sulfate (*r*^*2*^ = 0.36).’
6. The original version of this Article contained errors in Figure 2. The calculation of the variance explained was done incorrectly. Figure 2 has been updated with a corrected version. The legend of Figure 2, which incorrectly read ‘The variance in 1179 of the 1321 metabolites partly explained by variation in the gut microbiota from 8583 individuals aged 50 to 65 of the Swedish CArdioPulmonary bioImage Study.’ has been replaced by the correct sentence: ‘The variance in 1168 of the 1321 metabolites partly explained by variation in the gut microbiota from 8583 individuals aged 50 to 65 of the Swedish CArdioPulmonary bioImage Study.’
7. The original version of this Article contained errors in Supplementary Data 2 and 10. The calculation of the percentage of metabolite values detected above the detection threshold was done incorrectly. Supplementary Data 2 and 10 have both been updated with a corrected version.
8. The original version of this Article contained errors in Supplementary Data 5 and 10. The calculation of the variance explained was done incorrectly. Both Supplementary Data 5 and 10 have been updated with corrected versions.
9. The original version of this Article contained an error in the Methods, which incorrectly read ‘Participants received a pre-packaged fecal sample collection kit (barcoded tubes, gloves, Ziploc bags, and a paper collection bowl) including instructions on how to collect the sample at home. The participants were asked to store the samples at –20 °C in the home freezer until the study site visit.’ The correct version replaces these sentences with ‘At the first visit, participants received a pre-packaged fecal sample collection kit (barcoded tubes, gloves, Ziploc bags, and a paper collection bowl) including instructions on how to collect the sample at home. The participants were asked to store the samples at –20 °C in the home freezer until the study site visit. Of the 8538 samples that were part of this study, 8131 samples were returned at visit 2 (Uppsala, *n* = 4629; Malmö, *n* = 3502), 221 (Uppsala, *n* = 107; Malmö, *n* = 114) samples were received within a week after visit 2, and 139 (Uppsala, *n* = 31; Malmö, *n* = 108) 8 days or later after visit 2.’

All the errors have been corrected in both the PDF and HTML versions of the article.

## Supplementary information

Updated Supplementary Data 2

Updated Supplementary Data 5

Updated Supplementary Data 10

Incorrect Supplementary Data 2

Incorrect Supplementary Data 5

Incorrect Supplementary Data 10

