# Supplementary material for: An online atlas of human plasma metabolite signatures of gut microbiome composition
Source: Nat Commun. 2022 Sep 23;13:5370. doi: 10.1038/s41467-022-33050-0 (PMC9508139; doi:10.1038/s41467-022-33050-0)
Supplement: Supplementary file 1 — Supplementary Information [file 41467_2022_33050_MOESM1_ESM.pdf]

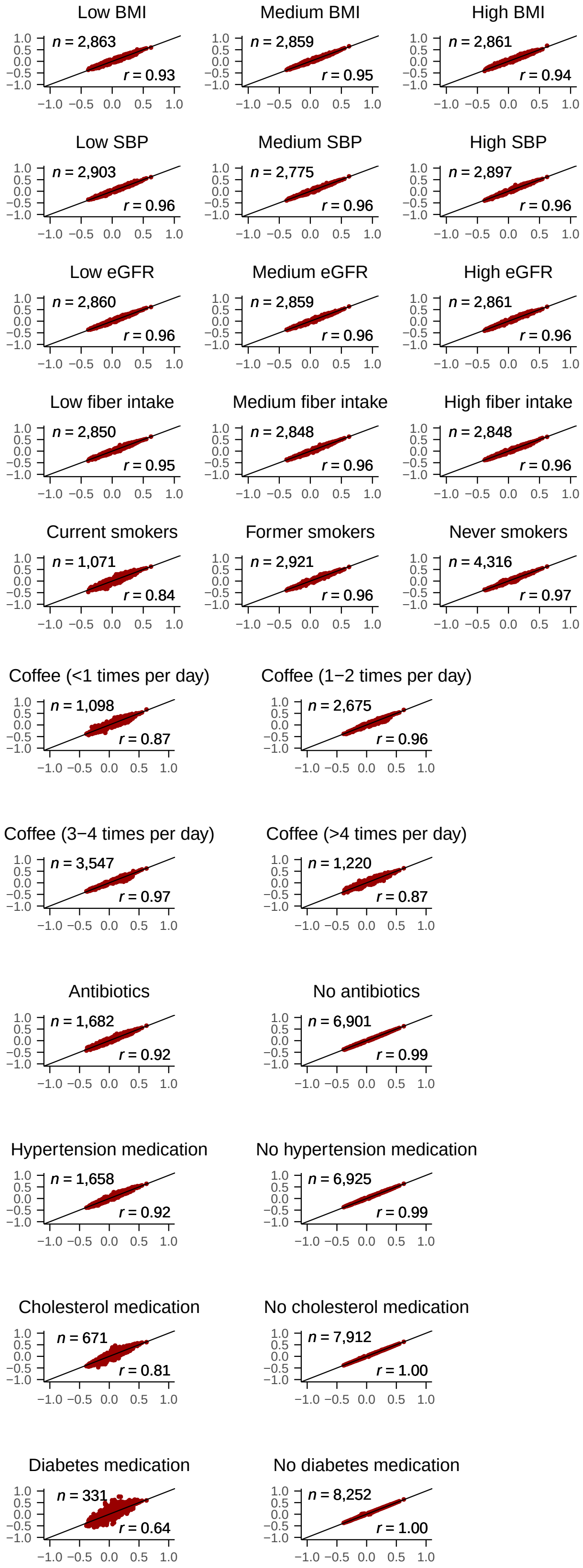

Supplementary Figure 1. Model for all participants compared to models stratified for potential confounders and mediators for 546,819 significant associations. X-axis, Spearman's ρ for the base model of 8,583 individuals; y-axis, Spearman's ρ for models stratified for BMI, body mass index; SBP, systolic blood pressure; Fiber intake, adjusted for energy intake; Antibiotics, antibiotics prescribed in the year before sampling; eGFR, estimated glomerular filtration rate calculated with the CKD-EPI Study equation (doi: 10.7326/0003-4819-150-9-200905050-00006); Coffee intake, self-reported;

Hypertension, cholesterol and diabetes medication, self-reported medication last 2 weeks. Plotted are the 1000 most extreme Spearman's ρ for each stratified model and a random sample of 50,000. Source data are provided as a Source Data file.

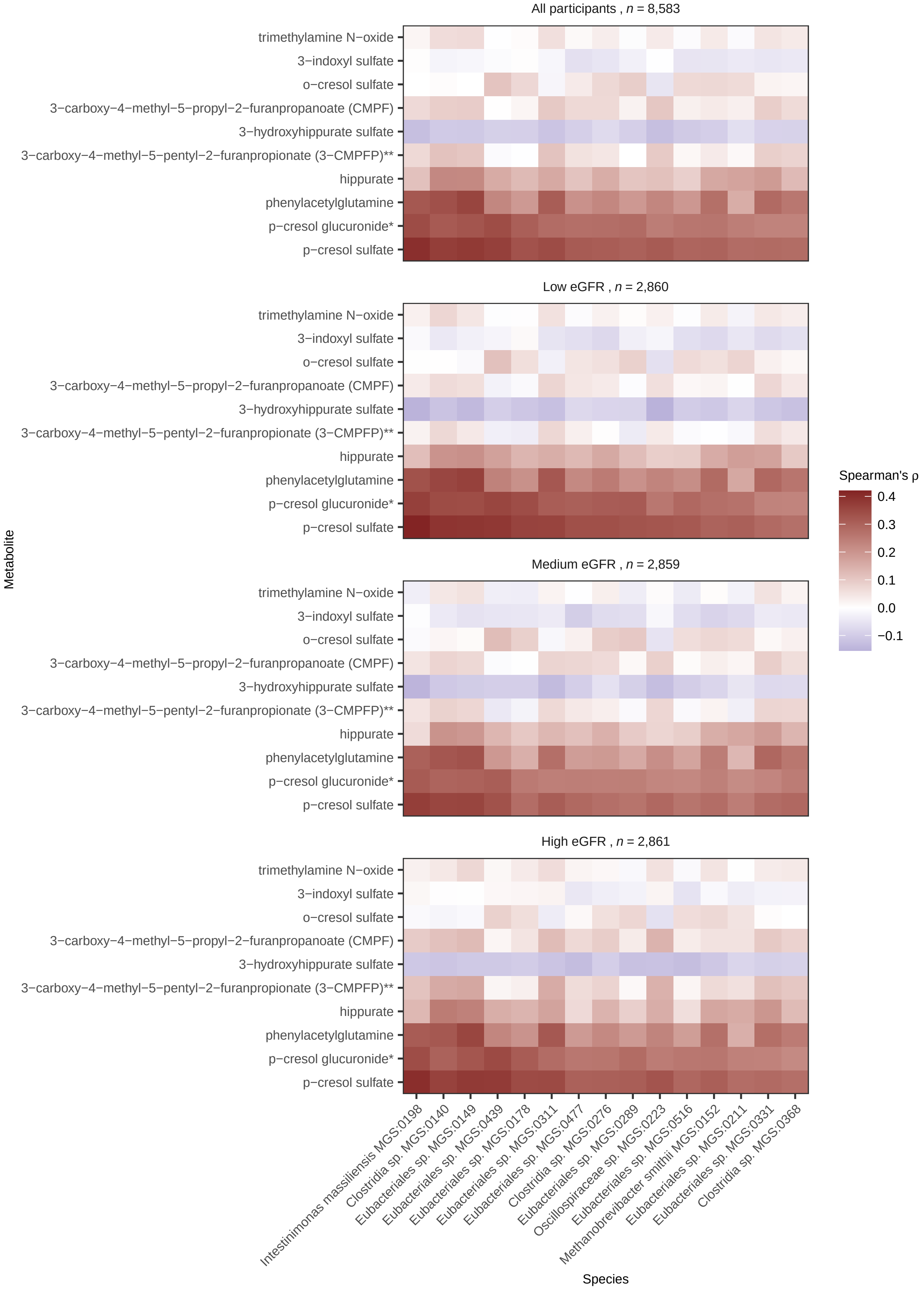
All participants , n = 8,583
Low eGFR , n = 2,860
Medium eGFR , n = 2,859
High eGFR , n = 2,861
trimethylamine N−oxide
3−indoxyl sulfate
o−cresol sulfate
3−carboxy−4−methyl−5−propyl−2−furanpropanoate (CMPF)
3−hydroxyhippurate sulfate
3−carboxy−4−methyl−5−pentyl−2−furanpropionate (3−CMPFP)**
hippurate
phenylacetylglutamine
p−cresol glucuronide*
p−cresol sulfate
Metabolite
Spearman's ρ
0.4
0.3
0.2
0.1
0.0
−0.1
Intestinimonas massiliensis MGS:0198
Clostridia sp. MGS:0140
Eubacteriales sp. MGS:0149
Eubacteriales sp. MGS:0439
Eubacteriales sp. MGS:0178
Eubacteriales sp. MGS:0311
Eubacteriales sp. MGS:0477
Clostridia sp. MGS:0276
Eubacteriales sp. MGS:0289
Oscillospiraceae sp. MGS:0223
Eubacteriales sp. MGS:0516
Methanobrevibacter smithii MGS:0152
Eubacteriales sp. MGS:0211
Eubacteriales sp. MGS:0331
Clostridia sp. MGS:0368
Species

Supplementary Figure 2. 15 microbial species most strongly correlated (based on *p*-value) with *p*-cresol sulfate and its association with *p*-cresol sulfate and other uremic toxins, non-stratified and stratified by kidney function assessed by estimated glomerular filtration rate, eGFR. The associations were assessed using partial Spearman's rank correlation between 1,528 gut microbial species and 10 candidate uremic toxins adjusted for age, sex, place of birth, study site, microbial DNA extraction plate and metabolomics delivery batch. Specific considerations: * and ** denotes metabolites annotated without an internal standard; eGFR were estimated with the CKD-EPI Study equation (doi: 10.7326/0003-4819-150-9-200905050-00006). Source data are provided as a Source Data file.

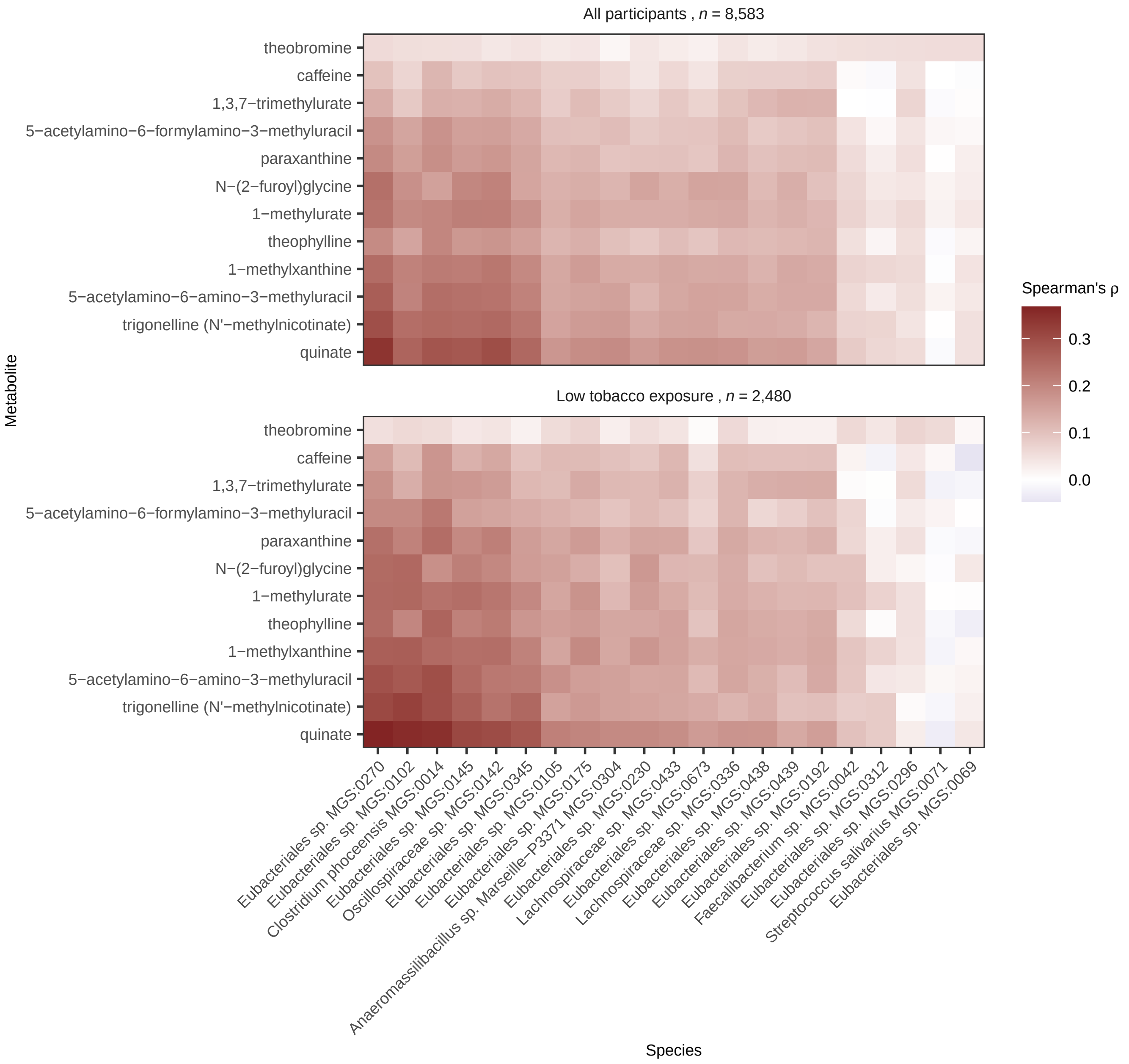

Supplementary Figure 3. Previously reported coffee-associated biomarkers and their association with microbial species in the full dataset (n = 8,583) and in 2,480 individuals who had low tobacco exposure (never smoked, no snus, no other nicotine products, no smokers in house and no smokers in the work environment). Partial Spearman's rank correlations were calculated for 1,528 gut microbial species and 1,321 plasma metabolites adjusted for age, sex, place of birth, study site, microbial DNA extraction plate and metabolomics delivery batch. Coffee biomarkers were selected based on studies by Shi et al. (doi: 10.1111/joim.13009) and Rothwell et al. (doi: 10.1371/journal.pone.0093474) For each coffee-associated metabolite, the 8 strongest associations with microbial species were selected based on their p-value, and the unique subset of those 21 species is shown. Source data are provided as a Source Data file.
