# Supplementary material for: An online atlas of human plasma metabolite signatures of gut microbiome composition
Source: Nat Commun. 2022 Sep 23;13:5370. doi: 10.1038/s41467-022-33050-0 (PMC9508139; doi:10.1038/s41467-022-33050-0)
Supplement: Supplementary file 2 — Description of Additional Supplementary Files [file 41467_2022_33050_MOESM2_ESM.docx]

Description of Additional Supplementary Files

An online atlas of human plasma metabolite signatures of gut microbiome composition. Dekkers et al.

File name: Supplementary Data 1

Description:

Supplementary Data 1. Taxonomic annotation of 1,528 metagenomic species

File name: Supplementary Data 2

Description:

Supplementary Data 2. Annotation of 1,321 plasma metabolites

File name: Supplementary Data 3

Description:

Supplementary Data 3. Baseline characteristics of the study participants from all six study sites, total study sample from SCAPIS-Uppsala and SCAPIS-Malmö, and the present study sample

File name: Supplementary Data 4

Description:

Supplementary Data 4. Association between Shannon diversity index and plasma metabolites

File name: Supplementary Data 5

Description:

Supplementary Data 5. Variance explained of plasma metabolite levels by variation in the gut microbiota

File name: Supplementary Data 6

Description:

Supplementary Data 6. Associations between gut microbial species and plasma metabolites

File name: Supplementary Data 7

Description:

Supplementary Data 7. Enrichment for metabolite subclasses in alpha diversity associations with metabolites

File name: Supplementary Data 8

Description:

Supplementary Data 8. Enrichment for metabolite subclasses in single metagenomic species associations with metabolites

File name: Supplementary Data 9

Description:

Supplementary Data 9. Enrichment for GMM modules in single metagenomic species associations with metabolites

File name: Supplementary Data 10

Description:

Supplementary Data 10. Summary of results for uremic toxins, omeprazole, metformin and coffee metabolites

File name: Supplementary Data 11

Description:

Supplementary Data 11. Comparison of plasma uremic toxin levels and kidney function

File name: Supplementary Data 12

Description:

Supplementary Data 12. Comparison of plasma coffee metabolite levels and coffee intake
