# Supplementary material for: An online atlas of human plasma metabolite signatures of gut microbiome composition
Source: Nat Commun. 2022 Sep 23;13:5370. doi: 10.1038/s41467-022-33050-0 (PMC9508139; doi:10.1038/s41467-022-33050-0)
Supplement: Supplementary file 15 — Reporting Summary [file 41467_2022_33050_MOESM15_ESM.pdf]

nature portfolio

Corresponding author(s): Tove Fall

Last updated by author(s): Aug 25, 2022

Reporting Summary

Nature Portfolio wishes to improve the reproducibility of the work that we publish. This form provides structure for consistency and transparency in reporting. For further information on Nature Portfolio policies, see our Editorial Policies and the Editorial Policy Checklist.

Statistics

For all statistical analyses, confirm that the following items are present in the figure legend, table legend, main text, or Methods section.

n/a	Confirmed	
☐	☒	The exact sample size (*n*) for each experimental group/condition, given as a discrete number and unit of measurement
☐	☒	A statement on whether measurements were taken from distinct samples or whether the same sample was measured repeatedly
☐	☒	The statistical test(s) used AND whether they are one- or two-sided<br>*Only common tests should be described solely by name; describe more complex techniques in the Methods section.*
☐	☒	A description of all covariates tested
☐	☒	A description of any assumptions or corrections, such as tests of normality and adjustment for multiple comparisons
☐	☒	A full description of the statistical parameters including central tendency (e.g. means) or other basic estimates (e.g. regression coefficient) AND variation (e.g. standard deviation) or associated estimates of uncertainty (e.g. confidence intervals)
☐	☒	For null hypothesis testing, the test statistic (e.g. *F*, *t*, *r*) with confidence intervals, effect sizes, degrees of freedom and *P* value noted<br>*Give P values as exact values whenever suitable.*
☒	☐	For Bayesian analysis, information on the choice of priors and Markov chain Monte Carlo settings
☒	☐	For hierarchical and complex designs, identification of the appropriate level for tests and full reporting of outcomes
☐	☒	Estimates of effect sizes (e.g. Cohen's *d*, Pearson's *r*), indicating how they were calculated

Our web collection on statistics for biologists contains articles on many of the points above.

Software and code

Policy information about availability of computer code

Data collection	For the collection of the metagenomic sequencing data the following software was used: Bowtie 2 v02.3.2, MEGAHIT v. 1.1.1, BWA mem v 0.7.16a and EggNOG-mapper v2.0.1.
Data analysis	All analyses were performed using R v4.1.1. R packages used: vegan v2.5-7, ppcor v1.1, glmnet v4.1-3 and fgsea v1.19.2 . Code related to the analyses in this study are available at https://github.com/MolEpicUU/GUTSY_Atlas.

For manuscripts utilizing custom algorithms or software that are central to the research but not yet described in published literature, software must be made available to editors and reviewers. We strongly encourage code deposition in a community repository (e.g. GitHub). See the Nature Portfolio guidelines for submitting code & software for further information.

Data

Policy information about availability of data

All manuscripts must include a data availability statement. This statement should provide the following information, where applicable:

- Accession codes, unique identifiers, or web links for publicly available datasets
- A description of any restrictions on data availability
- For clinical datasets or third party data, please ensure that the statement adheres to our policy

De-hosted anonymized metagenomic sequencing data generated in this study have been deposited in the European Nucleotide Archive under accession number PRJEB51353 (https://www.ebi.ac.uk/ena/browser/view/prjeb51353). Metabolomics analysis was performed at Metabolon, TX, USA, who deposited spectral data from the first analytical stage (MS1) for 125 anonymized samples from SCAPIS-Uppsala in MetaboLights under accession number MTBLS407 (https://www.ebi.ac.uk/metabolights/MTBLS407). However, MS/MS spectral data are not shared by Metabolon to the research community. Additional individual-level data are available under restricted access as they contain sensitive personal information that are protected under privacy laws, and access can be obtained following ethical approval

March 2021

from the Swedish Ethical Review Board (https://etikprovningsmyndigheten.se/; the application procedure and instructions are provided in the link) and data access approval from the SCAPIS Data access board (https://www.scapis.org/data-access/; the application procedure and conditions are provided in the link). These data may only be used for research, and are not available for commercial use. Underlying data for all figures are provided in the Source Data file and available at https://github.com/MolEpicUU/GUTSY_Atlas. A companion website to the article containing the full results of the current study and further study-related searchable material can be accessed via https://gutsyatlas.serve.scilifelab.se/.

Field-specific reporting

Please select the one below that is the best fit for your research. If you are not sure, read the appropriate sections before making your selection.

- [x] Life sciences
- [ ] Behavioural & social sciences
- [ ] Ecological, evolutionary & environmental sciences

For a reference copy of the document with all sections, see nature.com/documents/nr-reporting-summary-flat.pdf

Life sciences study design

All studies must disclose on these points even when the disclosure is negative.

Sample size	All SCAPIS Uppsala and Malmö samples with complete metagenomic sequencing data, plasma metabolite profiling data and main model covariates were used (n = 8,583). No power calculations were done, but similar previous studies with a fraction of the samples were successful in finding significant associations.
Data exclusions	During the metagenomic sequencing measurement, 12 samples were excluded as they provided a low DNA yield and/or had features interfering with the library preparation even after one attempt of re-extraction each. Furthermore, one sample with only 1,473 reads mapped to the signature genes was removed after sequencing. For the metabolomics profiling, two samples were lost during processing, two samples were excluded whose data were determined to be outliers based on principal component analysis, and one sample was excluded based on clearly discordant levels of glucose, cholesterol and creatinine compared to the reference laboratory measurement. Finally, 33 individuals with missing main model covariates were removed. Species with at least 100 non-zero measurements and metabolites with at least 100 values above the detection threshold were included in the present study. These exclusion/inclusion criteria were established before analyses were performed.
Replication	We did not include any new replication data.
Randomization	This is not relevant to our study, because we did not perform any group based analysis.
Blinding	This is not relevant to our study, because we did not perform any group based analysis.

Reporting for specific materials, systems and methods

We require information from authors about some types of materials, experimental systems and methods used in many studies. Here, indicate whether each material, system or method listed is relevant to your study. If you are not sure if a list item applies to your research, read the appropriate section before selecting a response.

Materials & experimental systems

n/a	Involved in the study	
x		Antibodies
x		Eukaryotic cell lines
x		Palaeontology and archaeology
x		Animals and other organisms
	x	Human research participants
x		Clinical data
x		Dual use research of concern

Methods

n/a	Involved in the study	
x		ChIP-seq
x		Flow cytometry
x		MRI-based neuroimaging

Human research participants

Policy information about studies involving human research participants

Population characteristics	Table 1 describes the main sociodemographic and clinical characteristics of the Malmö and Uppsala SCAPIS study sites included in the current study. Included are; age, sex, place of birth, body mass index, systolic blood pressure, estimated glomerular filtration rate, current smoking status, fiber intake, coffee intake, antibiotics prescriptions, blood pressure, lipid-lowering and diabetes medication, and metformin and omeprazole prescriptions.
Recruitment	SCAPIS is a prospective population-based study of 30,154 men and women, aged 50–65 years, living in six municipality regions in Sweden. It was designed with the main aim to improve risk prediction and understanding of cardiovascular disease, chronic pulmonary obstructive disease, and related metabolic disorders. After a pilot study in 2012, recruitment was initiated in 2014 and completed in 2018. Individuals were randomly recruited from the population register, with a participation rate of 50%. The present study is based on the data for 11,287 participants from the test centers in Malmö (n = 6,251) and Uppsala (n = 5,036).<br><br>From Bergström et al. Journal of Internal Medicine (2015):<br>Recruitment<br>All Swedish residents have a unique personal identification number (PIN), which allows unbiased and randomized recruitment from the Swedish population register, almost complete follow-up, and linkage to registries with information on hospitalizations, living conditions and social welfare (www.socialstyrelsen.se/statistics, www.scb.se/ en_/). Initial contact is made by sending out an informational brochure asking the recipient to contact the study centre via telephone, e-mail or letter. If the centre is not contacted, the recipient is reminded by up to three telephone calls (including one in the evening) and finally by letter. If the centre is contacted and the subject is willing to participate in the study, an appointment is arranged at the study centre. No exclusion criteria are applied except the inability to understand written and spoken Swedish for informed consent. No reimbursement is provided for travel expenses or loss of income. To improve recruitment, the study is advertised in local newspapers and on television. Employers in the catchment areas are targeted to encourage study participation with paid leave.<br>Participation rates [in pilot of 1,111 individuals]<br>The overall participation rate was 49.5% (39.9% and 67.8% in areas of low and high socio-economic status, respectively). Reasons for nonparticipation were as follows: inability to make contact with the subject (37.4%), too busy (15.7%), too sick (6.6%), language difficulties (7.8%), miscellaneous (6.4%) and none given (26.1%). Lack of contact and language difficulties dominated in areas of low socio-economic status.<br><br>We think this has a limited effect on our results.
Ethics oversight	All study participants provided a signed informed consent at the first site visit. The study adheres to the Declaration of Helsinki and was approved by the Swedish Ethics Review Authority (Etikprövningsmyndigheten Dnr 2010-228-31M, Dnr 2018-315).

Note that full information on the approval of the study protocol must also be provided in the manuscript.
